# Discovery of novel peptidomimetics against HSP90–HOP interactions towards improved cancer therapeutics using machine learning strategies

**DOI:** 10.3389/fbinf.2026.1847387

**Published:** 2026-07-10

**Authors:** Sarath Perumal, Ramanathan Karuppasamy

**Affiliations:** Department of Biotechnology, School of Bio Sciences and Technology Vellore Institute of Technology, Vellore, Tamil Nadu, India

**Keywords:** classification model, ensemble docking, HSP90–HOP interface, machine learning-based scoring functions, molecular dynamics simulation, peptidomimetics, statistical significance

## Abstract

**Background:**

The HSP90–HOP interaction orchestrates transfer of client proteins from HSP70 to HSP90, promoting their conformational maturation and stabilisation and thereby sustaining oncogenic signalling. The study explores a promising alternative for cancer chemotherapy by targeting this interface rather than the traditional ATP-binding site, which may circumvent the toxicity associated with classical HSP90 inhibitors. Despite its therapeutic relevance, the HSP90–HOP interface remains underexplored, particularly in the context of structure-guided peptidomimetic inhibitors, highlighting a critical gap in strategies to modulate proteostasis in cancer.

**Aim:**

This study sought to identify a promising peptidomimetic molecule capable of disrupting the HSP90–HOP interface.

**Methods:**

A seven-residue template peptide was engineered from a crucial segment of HOP, with hotspot residues identified through *in silico* mutagenesis. These residues were subsequently employed to retrieve a library of 200 peptidomimetic molecules. An in-house developed classification-based machine learning model served as the primary screening tool to identify potential HSP90–HOP interaction modulators. The shortlisted compounds were subsequently evaluated by molecular docking, binding free energy estimation, machine learning-assisted scoring, and ADMET profiling to ensure structural stability, binding reliability, and pharmacokinetic suitability. This scrutiny resulted in the selection of five lead compounds, with MMs01053537 emerging as a top candidate.

**Results:**

The ML model achieved an accuracy of 0.9055 and an ROC-AUC of 0.9537, indicating strong predictive performance of the model. The lead molecule MMs01053537 demonstrated a favourable binding score of −85.92 kcal/mol, along with a robust stability profile during molecular dynamics simulations. To further assess the consistency of the predicted binding mode across trajectory-derived conformations, ensemble docking and MD-enhanced binding free-energy analysis, along with statistical evaluation were performed.

**Conclusion:**

Collectively, these findings position MMs01053537 as a potential candidate for disrupting the HSP90–HOP interaction. However, experimental validation remains essential to confirm its therapeutic potential and support further biological evaluation of the compound.

## 1 Introduction

Heat Shock Protein 90 (HSP90) is indispensable for the post-translational folding, stabilization, and activation of a diverse array of client proteins, including cyclin-dependent kinase 4 (CDK4), epidermal growth factor receptor (EGFR), and vascular endothelial growth factor receptor (VEGFR). The activity is regulated by co-chaperones, among which the HSP70-HSP90 organizing protein (HOP) occupies a central position (Okpara et al., 2024). Recognizing the critical role of HSP90 in cancer, researchers initially focused on developing N-terminal ATP antagonists. Traditionally derived *N*-terminal ATP antagonists of HSP90 have shown initial promise but are hindered by several limitations, such as toxicity and narrow therapeutic indices, thereby impeding their clinical development (Sumi and Ghosh, 2022). Therefore, to get around these constraints, the focus has turned to protein-protein interaction modulators that specifically disrupt co-chaperone assembly in the region of HSP90. This targeted approach presents a precise and efficacious anti-cancer strategy with reduced systemic toxicity observed with conventional inhibitors such as geldanamycin (Schwarz et al., 2022). Thus, targeting the interaction between HSP90 and HOP offers a promising therapeutic approach that blocks the transfer of cancer-promoting clients without directly affecting the ATPase function of HSP90.

Despite the emergence of several strategies aimed at disrupting the HSP90–HOP interaction, currently available inhibitor classes, including small molecules and peptide-based modulators, have shown important limitations related to pharmacokinetic properties, selectivity, or insufficient experimental validation. Yi and Regan (2008) identified six 7-azapteridine derivatives that effectively disrupt the C-terminal interaction between HSP90 and HOP. Despite potent *in vitro* activity, they lack *in vivo* efficacy and suboptimal pharmacokinetic profiles. In another study, Gupta et al. (2013) reported PEP73 to be potent in disrupting the HSP90–HOP interaction; however, a lack of *in vitro* validation and subsequent studies have not been reported yet. Furthermore, a novel small-molecule inhibitor, Y-632, was identified by Wang et al. (2016), which also disrupted the interaction however, it lacks detailed toxicology studies. Given the limitations of existing approaches to inhibit the HSP90–HOP interaction, there is a pressing need for novel therapeutics addressing pharmacokinetic and toxicological concerns to advance potential candidates for clinical application.

**FIGURE 1:**
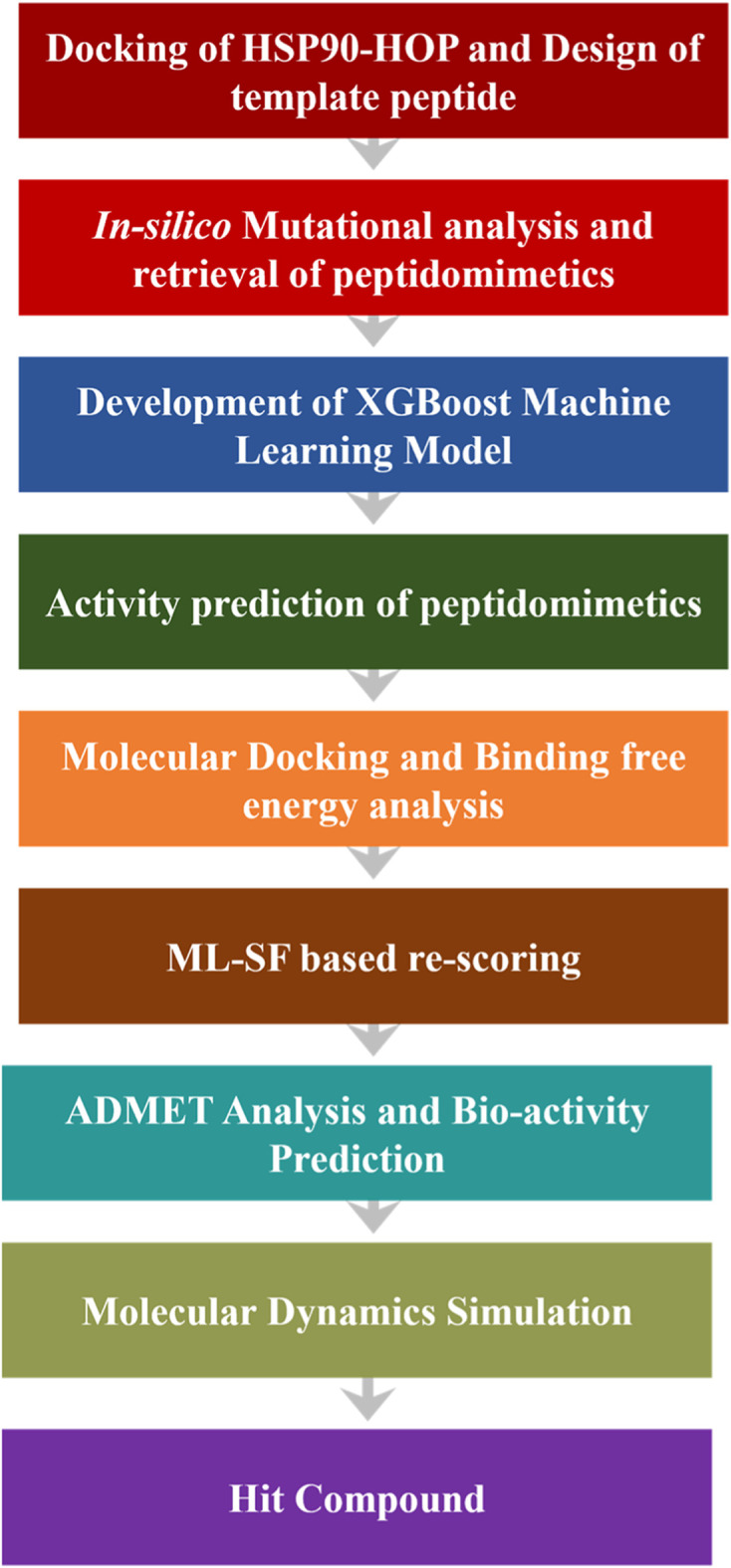
Schematic workflow of the study, illustrating the computational screening pipeline, integrated with machine learning-based prediction of active compounds, followed by virtual screening and ending with the prioritisation of the final hit compound.

Peptidomimetics offer a compelling strategy for targeting PPIs by overcoming the above limitations. The physicochemical and structural properties of peptidomimetics allow them to better mimic the extended binding patterns observed in natural protein interfaces. Moreover, peptidomimetics provide greater conformational adaptability compared with rigid small molecules. This flexibility allows them to conform to shallow or dynamic interfaces and maintain favourable interactions with multiple residues across the protein surface (Lombardi et al., 2025). Therefore, we propose the development of novel HOP resembling peptidomimetic inhibitors that competitively bind to HSP90. The schematic representation of the work flow is depicted in Figure 1. This approach holds promise for a new class of anti-cancer agents that precisely target the chaperone machinery at the co-chaperone interface, addressing a critical gap in current HSP90-directed therapies.

## 2 Materials and methods

### 2.1 Prediction of binding site

The study utilised the crystal structures of HSP90 (PDB ID: 2K5B) and HOP (PDB ID: 1 E LR), obtained from the RCSB Protein Data Bank (PDB). Since a complete experimental co-crystal structure of the HSP90–HOP complex for the selected domains was not available, the potential binding pocket at the HSP90–HOP interaction interface was predicted using the SiteMap module of Schrödinger (Schrödinger, LLC, New York, NY). The prepared HSP90–HOP complex structure was imported into Maestro and processed using the Protein Preparation Wizard to assign bond orders, add hydrogen atoms, optimise hydrogen-bonding networks, and minimise the structure under the OPLS force field. SiteMap analysis was then performed on the HSP90 surface to identify and rank probable binding pockets within and around the interface region involved in HOP recognition (Halgren, 2009). The SiteMap algorithm evaluates pocket geometry and physicochemical properties, including enclosure, solvent exposure, hydrophobicity/hydrophilicity balance, and hydrogen-bonding potential. It assigns each site a Druggability score (DScore) to estimate binding-site suitability and druggability. The top-ranked pocket, based on the highest DScore and its proximity to the HOP-interacting interface residues, was selected as the most plausible site for subsequent analyses.

### 2.2 Protein-protein docking analysis

The HSP90-HOP interaction complex was generated through protein–protein docking using the pyDockWEB server (https://life.bsc.es/pid/pydockweb). The docking poses are produced using a rigid-body docking approach and subsequently evaluated using a scoring function that incorporates electrostatic interactions, desolvation energy, and limited van der Waals contributions to identify energetically favourable binding conformations (Jiménez-García et al., 2013). The top-ranked docked complex, based on the pyDock scoring function, was selected for subsequent analysis. The final docking score represents a comparative interaction energy metric, with more negative values indicating more favourable predicted docking conformations. The docking and scoring outputs presented in the manuscript report far more negative than typical experimentally measured small-molecule binding free energies. Therefore, these values are commonly used for relative ranking rather than affinity quantification, as it is not calibrated to the physical free energy value. The overall pyDock scoring function can be represented as:

$$E_{\text{total}}=E_{\text{elec}}+E_{\text{desolv}}+w\times E_{\text{vdW}}$$

where

E_total_ -final pyDock docking score

E_elec_ -electrostatic interaction energy

E_desolv_-desolvation energy

E_vdW_-van der Waals interaction energy

w-weighting factor applied to the VdW term

### 2.3 Design of template peptide

The binding interface of the HSP90–HOP complex was analysed by identifying residues within a 4 Å interaction distance in the complex to design the template peptides. This was followed by modelling the peptide using the “Build Structure” tool in UCSF Chimera (Pettersen et al., 2004). Computational predictions substantially reduce the need for extensive experimental screening by highlighting the most impactful mutations. Therefore, the key residues of the designed peptide were identified through site-directed mutagenesis using the Rotamer tool in UCSF Chimera (Moreira et al., 2007). The designed peptide and mutant structures were then minimised using the Amber ff14SB force field in UCSF Chimera and subsequently docked with HSP90 using the pyDockWEB algorithm (https://life.bsc.es/pid/pydockweb).

### 2.4 Retrieval of peptidomimetics

The pepMMsMIMIC server (http://mms.dsfarm.unipd.it/pepMMsMIMIC/) was employed to retrieve potential peptidomimetics based on the selected key residues of the designed peptide. The hybrid scoring function combining 60% pharmacophoric similarity and 40% shape similarity was chosen from the scoring-method menu to guide the retrieval of structurally and functionally relevant mimetic candidates (Floris et al., 2011).

### 2.5 Development of machine learning model

#### 2.5.1 Dataset preparation

A curated dataset of experimentally reported active compounds targeting the HSP90 was retrieved from ChEMBL, which consists of 766 active compounds (Zdrazil, 2025). To construct a balanced binary classification model, 766 inactive compounds were retrieved from Schrödinger. The final dataset therefore, contained 1,532 compounds comprising equal numbers of active and inactive molecules. Physicochemical and ADME-related molecular descriptors were generated using the QikProp module of Schrödinger. Descriptors containing missing or invalid values were removed before model development.

#### 2.5.2 Model development

Traditional supervised ML approaches were employed for the binary classification of active and inactive compounds. The descriptor matrix was processed using machine learning libraries, which included Scikit-learn and XGBoost. The target labels were assigned as: Active compounds = 1 and Inactive compounds = 0. The complete dataset was divided into training and testing subsets using a stratified random split with an 80:20 ratio to preserve class balance across both subsets. To reduce dimensionality and eliminate redundant descriptors, feature selection was performed using the SelectKBest algorithm with mutual information classification scoring (Vergara and Estévez, 2014). The top 40 informative descriptors exhibiting the highest discriminatory power toward the activity label were retained for model construction.

Extreme Gradient Boosting (XGBoost) was utilised as a predictive model due to its strong predictive performance, scalability, and capability to capture complex nonlinear relationships within high-dimensional molecular descriptor datasets. XGBoost employs an ensemble boosting framework with regularisation mechanisms that improve model robustness while minimising overfitting, thereby making it highly suitable for cheminformatics and bioactivity prediction studies (Sheridan et al., 2016). Furthermore, our research team developed and benchmarked multiple classification models, among which XGBoost consistently demonstrated superior predictive performance across several evaluation metrics (Antony and Karuppasamy, 2023; Premnath et al., 2026). Alongside, recent studies have reported the successful application of XGBoost in virtual screening, molecular property prediction, ADMET modelling, and drug discovery-related classification tasks (Nadjafi-Arania et al., 2026; Yang et al., 2023). Further, model tuning was performed (Table 1). Model performance was evaluated using multiple statistical metrics, including Accuracy, Receiver Operating Characteristic Area Under the Curve (ROC-AUC), Precision–Recall Area Under the Curve (PR-AUC), Matthews Correlation Coefficient (MCC), F1-score, and Confusion matrix analysis. The optimised model was retrained on the complete dataset and applied to the external peptidomimetic dataset to predict potential active compounds.

**TABLE 1:** Optimised hyperparameters employed for the development of the XGBoost classification model.

Hyperparameter	Value
n_estimators	150
max_depth	3
learning_rate	0.05
Subsample	0.8
colsample_bytree	0.8

### 2.6 Docking of peptidomimetics

Subsequently, the peptidomimetics were docked with the receptor using Molsoft ICM Pro (MolSoft LLC, San Diego, CA, United States). The ICM scoring function combined van der Waals, hydrogen-bonding, electrostatic, solvation, ligand strain and torsional-entropy terms. The ligands were represented in internal coordinates and sampled using biased-probability Monte Carlo with local minimisation on precomputed receptor potential grids. The receptor flexibility was handled by multi-conformer (4D) docking and side-chain refinement (Neves et al., 2012)

### 2.7 Machine learning-based scoring functions

Several machine learning-based scoring functions (ML-SF), including KDEEP, SF-CNN, and X-Score, were utilised to revalidate the binding mechanism of peptidomimetics. KDEEP and Sf-CNN are pre-trained scoring functions based on a three-dimensional convolutional neural network (3D-CNN) for predicting protein–ligand binding affinity. In KDEEP, the protein–ligand complex is converted into a three-dimensional voxelized grid centred on the ligand, with each voxel encoding spatial chemical descriptors that capture the local physicochemical environment of the complex. The binding affinity is then estimated through the nonlinear transformation learned by the trained CNN model (Jiménez et al., 2018). Similarly, in SF-CNN, the protein–ligand complex is represented as a three-dimensional grid or tensor that encodes atom-type information within the binding pocket. Atomic environments are described using one-hot encoded atom-type channels, enabling the CNN to learn interaction patterns directly from the structural representation of the complex (Wang et al., 2022). X-Score is an empirical scoring function that represents terms for van der Waals interaction, hydrogen bonding, hydrophobic effect and deformation effect (Wang et al., 2002). It can be expressed as:

$$\Delta G_{\text{binding}}=\Delta G_{\text{vdw}}+\Delta G_{H-\text{bond}}+\Delta G_{\text{hydrophobic}}+\Delta G_{\text{rotor}}+\Delta G_{0}$$

These scoring functions provide an orthogonal, data-driven re-scoring of docked poses by learning patterns from experimental affinities, improving discrimination of true binders and exposing pose-dependent features that classical scores may miss (Isaac et al., 2024).

### 2.8 Binding free energy analysis

The binding free energy analysis was carried out using the PRODIGY-LIG no-electrostatics regression model (ΔG__noelec_) (Kurkcuoglu et al., 2018). The binding free energy is estimated by quantifying the interfacial contacts, such as atomic contacts and non-interacting surface areas of proteins and ligands. It is estimated by

$$\Delta G_{\text{noelec}}=0.0354707{\text{AC}}_{\text{NN}}-0.1277895{\text{AC}}_{\text{XX}}-0.0072166{\text{AC}}_{\text{CN}}-5.1923181$$

where,

NX - nitrogen–other contacts

XX - other–other contacts, including polar hydrogens and non-C/N/O atoms

CN - carbon–nitrogen contacts

The resulting ΔG values were interpreted as comparative contact-based affinity estimates rather than absolute thermodynamic free-energy measurements since the approach correlates structural interface descriptors with experimentally derived affinities to provide reliable binding free energy predictions (Vangone et al., 2019).

### 2.9 Drug-likeness screening with bioactivity prediction

The peptidomimetics were subsequently subjected to ADMET analysis using the Deep-PK server (https://biosig.lab.uq.edu.au/deeppk/), which uses deep neural network trained on large-scale ADME datasets to evaluate the drug-like properties (Myung et al., 2024). The biological activity profile of the hit compound was predicted using the PASS server (http://www.way2drug.com/PASS). This is an online tool that encodes molecules with Multilevel Neighbourhoods of Atoms (MNA) descriptors and applies a Bayesian classifier to estimate the probability of activity (Pa) and inactivity (Pi) across more than 4,000 biological endpoints (Filimonov et al., 2014).

### 2.10 Molecular dynamics simulation

Molecular dynamics (MD) simulations were conducted to assess the stability and conformational changes of protein-ligand complexes, employing the Gröningen Machine for Chemical Simulations (GROMACS) 2020.3 package with the CHARMM36 (Best et al., 2012) for protein–reference and protein-peptidomimetic complexes. The CGenFF server was used to generate the ligand parameters and topology files. The molecular complexes were solvated in a dodecahedral box using an explicit simple point charge (SPC) water model. The system was subsequently neutralised by adding 3 Na^+^ ions to the system, energy minimised, and equilibrated by eliminating the weak Van der Waals interactions through the steepest descent algorithm. The Particle Mesh Ewald method and the linear constraint solver algorithm (LINCS) were employed to evaluate covalent bond and electrostatic interactions. The canonical NVT (number of particles, volume, and temperature) and isobaric NPT (number of particles, pressure, and temperature) ensembles were employed to equilibrate the system over a 100 ps simulation period. The system was equilibrated by heating to 300 K over 0.1 ps at a pressure of 1 bar, using the Berendsen and Parrinello-Rahman coupling methods. The equilibrated systems were simulated for 100 ns? Parameters such as the root mean square deviation (RMSD), root mean square fluctuation (RMSF), solvent-accessible surface area (SASA), radius of gyration (Rg), and Gibbs free energy landscape (FEL) were analysed using the obtained trajectories (Suresh and Karuppasamy, 2025).

### 2.11 Ensemble docking and MD-enhanced binding free-energy analysis

Ensemble docking and MD-enhanced binding free-energy analyses were performed to evaluate the stability and robustness of the predicted HSP90 interaction profiles under dynamic conformational conditions. HSP90 conformations were extracted from the equilibrated regions of the molecular dynamics trajectory at selected time intervals (0, 20, 40, 60, 80, and 100 ns). Each extracted structure was prepared independently and used as a receptor ensemble for subsequent docking analyses. The reference compound and the selected hit compound were docked independently against each MD-derived receptor conformations using the PyDockWEB server and Molsoft ICM-Pro, respectively.

## 3 Results

### 3.1 Identification of binding sites

**FIGURE 2:**
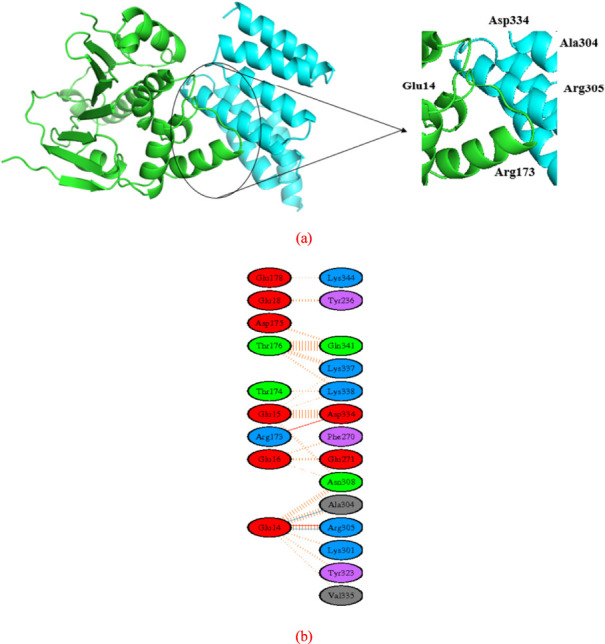
**(a)** The docked model of the human HSP90 (green) and HOP (blue) with crucial residues highlighted; **(b)** Interaction analysis for the top-ranked complex of the HSP90-HOP docked complex using PDBsum Generate

The SiteMap analysis identified multiple surface cavities on HSP90; however, the top-ranked pocket was localized within the interface region associated with HOP recognition. The identified site demonstrated a high DScore of 0.89, indicating a potentially druggable protein, which exhibited favourable geometric and physicochemical characteristics. Subsequent protein–protein docking using PyDock generated multiple HSP90–HOP complex conformations. Among the generated poses, pose 1 exhibited the highest docking score and showed strong spatial correspondence with the top-ranked SiteMap-predicted pocket. Further, interface residue analysis performed using PDBSum Generate revealed stable intermolecular interactions involving key interface residues within the internal region of HSP90, which are known to play a crucial role in HOP recognition and complex stabilisation. Notably, two residues on HSP90 (Glu14 and Arg173) and three residues on HOP (Ala304, Arg305 and Asp334) were identified as vital contributors to the stabilisation of the complex, as depicted in Figure 2a.

Importantly, the overlap between the SiteMap-predicted pocket and the top-ranked docking pose in PyDock indicates strong agreement between pocket prediction and interface docking analysis. These observations support the structural relevance of the identified binding region and suggest that the selected interface pocket may serve as a suitable target site for subsequent inhibitor design studies. This highlights the crucial role of these interactions in maintaining the complex’s stability.

### 3.2 Interface-driven design of peptidomimetic candidates

The continuous segment from Lys301 to Gly307 in HOP served as the foundation for obtaining a template peptide for the retrieval of peptidomimetic compounds. Given the crucial role of residues in complex stability, a 7-mer template peptide, p-KAYARIG, was designed. *In silico* mutational analysis of the peptide identified 200 peptidomimetic compounds.

### 3.3 Evaluation of machine learning model

#### 3.3.1 Model performance

**FIGURE 3:**
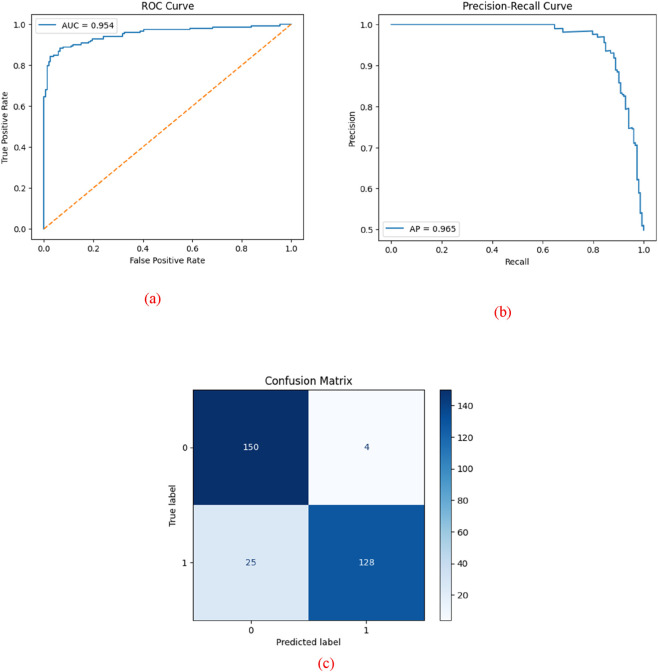
Performance evaluation of the XGBoost model on the test set: **(a)** ROC curve demonstrating strong discrimination between active and inactive compounds, **(b)** Precision–Recall curve indicating high predictive precision and recall, and **(c)** confusion matrix showing robust classification performance with low false-positive and false-negative predictions.

The balanced dataset minimised classification bias and enabled robust binary prediction modelling. QikProp descriptor generation produced a comprehensive physicochemical feature matrix representing molecular topology, lipophilicity, polarity, permeability, and drug-likeness characteristics. The optimised XGBoost classifier demonstrated high predictive capability on the independent test dataset, achieving an accuracy of 0.9055, ROC-AUC: 0.9537, PR-AUC: 0.9649, MCC: 0.8187 and F1-Score: 0.8982. The ROC curve demonstrated excellent discrimination between active and inactive compounds, indicating strong sensitivity and specificity across classification thresholds (Figure 3).

#### 3.3.2 Activity prediction of peptidomimetics

The final optimised model was subsequently applied to the external peptidomimetic dataset. 127 compounds demonstrated high predicted probability scores for activity against the HSP90, indicating their potential as promising lead candidates for further molecular docking analysis

### 3.4 Molecular docking and re-scoring functions

Geldanamycin and its derivative, 17-AAG, both established N-terminal inhibitors, along with celastrol and withaferin A, known C-terminal inhibitors, were used as reference compounds for comparative evaluation. The binding free energies of geldanamycin and 17-AAG were calculated to be −5.79 kcal/mol and −5.76 kcal/mol, respectively. Among the C-terminal inhibitors, celastrol and withaferin A exhibited binding free energy of −4.23 kcal/mol and −4.29 kcal/mol, respectively. A total of 127 peptidomimetic compounds predicted as active compounds were subsequently subjected to molecular docking and binding free energy analysis. 64 candidates exhibited more favourable binding free energy values and were selected for further evaluation based on comparison with the reference compounds. These shortlisted compounds were then assessed using the ML-SF, in which 49 candidates demonstrated better scores relative to the reference compounds. Of note, the compound selection was based on relative ranking and consensus performance across docking, binding free energy and Machine Learning (ML) assisted rescoring rather than on a universal absolute cutoff threshold, since such thresholds are method-dependent and vary across scoring functions. Accordingly, ML rescoring was employed as a consensus-based prioritisation strategy to refine docking-derived selection rather than as an independent classification threshold.

### 3.5 Prediction of ADMET properties

**FIGURE 4:**
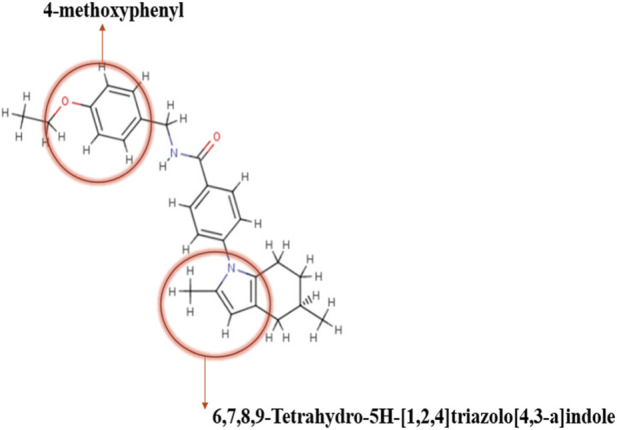
The hit peptidomimetic compound (MMs01053537) underlining its pharmacologically relevant moieties.

A total of 42 compounds complied with Lipinski’s Rule of Five, indicating drug-like properties. Bioavailability and intestinal absorption were further evaluated as critical pharmacokinetic parameters to estimate systemic exposure and therapeutic efficacy of orally administered candidates. Only 12 compounds out of 42 compounds demonstrated high predicted bioavailability and efficient intestinal absorption, thus narrowing the selection of candidates with enhanced pharmacokinetic potential. These compounds were subjected to Drug-Induced Liver Injury (DILI) prediction to further ensure the safety profile. Of these, only five were predicted to be non-DILI-causing, indicating a significantly reduced risk of hepatotoxicity. These five non-toxic, pharmacokinetically favourable molecules were shortlisted as lead compounds for further validation. The results are depicted in Tables 2, 3. MMs01053537 emerged as the most promising candidate among the five shortlisted lead compounds, as it exhibits interaction with key residues in HSP90. We analysed the structural backbone of the hit molecule to elucidate its inhibitory mechanism and efficacy against HSP90. The crucial moieties present in our hit molecules include 4-methoxyphenyl and 6,7,8,9-Tetrahydro-5H-[1,2,4] triazolo [4,3-a] indole, as illustrated in Figure 4. The hit molecule also exhibited a substantial Pa value of 0.452 and a considerably lower Pi of 0.032, suggesting a strong capability to act as an HSP27 antagonist.

**TABLE 2:** Docking scores and binding free energy of top candidates, along with the reference compounds.

S. NO	Compound ID	Docking score (kcal/mol)	Binding free energy (kcal/mol)
1	Geldanamycin	−74.24	−5.79
2	17-AAG	−70.69	−5.76
3	Celastrol	−64.12	−4.23
4	Withaferin A	−69.01	−4.29
5	MMs00894984	−28.76	−6.05
6	MMs01053537	−85.92	−7.11
7	MMs02731053	−70.48	−6.58
8	MMs02296444	−20.13	−5.87
9	MMs02296442	−34.74	−5.90

**TABLE 3:** Physiochemical properties of the top 5 candidates screened by the machine-learning integrated virtual screening pipeline.

S. NO	Compound ID	MWT^#^	CLog P	HbA	HbD	R.B	F.B^$^	T.C
1	MMs00894984	445.56	0.437	8	4	2	1.05	7.08
2	MMs01053537	402.53	4.239	4	1	7	2.65	8.48
3	MMs02731053	315.19	−1.221	3	5	2	0.21	1.25
4	MMs02296444	360.5	−0.324	4	4	2	1.17	6.88
5	MMs02296442	388.42	0.385	9	2	5	1.17	5.91

MWT- molecular weight; CLogP-Calculated partition coefficient; H_b_A-Hydrogen bond acceptor; H_b_D-Hydrogen bond donors; R.B- rotational bonds; F.B-Fraction unbound; T.C-Total clearance.

^#^ Expressed in Da.

^$^ Expressed in log ml/min/kg.

### 3.6 Molecular dynamics simulation

**FIGURE 5:**
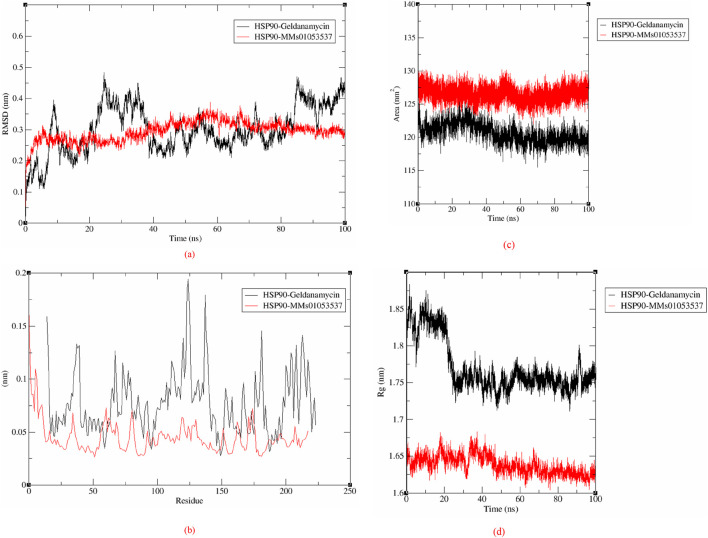
Molecular dynamics trajectory analysis of HSP90-Geldanamycin (reference) and HSP90-MM01053537 (hit) complexes, shown in black and red trendlines, respectively. **(a)** RMSD, **(b)** RMSF, **(c)** SASA, **(d)** Rg.

In the present molecular dynamics analysis, Geldanamycin was chosen as the reference compound with the identified hit molecule due to its superior performance in docking analysis relative to other modulators and its reported activities in prior studies as a potent HSP90 inhibitor. The black trendline corresponds to HSP90–geldanamycin complex, whereas the red trendline represents the HSP90–MMs01053537 complex. The RMSD profile of HSP90–geldanamycin complex exhibited greater conformational drift throughout the 100 ns trajectory. As seen in Figure 5a, the backbone RMSD initially increased from approximately 0.10–0.15 nm and exhibited pronounced fluctuations, peaking at about 0.45–0.48 nm around 20–35 ns and again toward the end of the simulation. Although the system did not exhibit catastrophic instability, the repeated excursions indicate a less uniform conformational behaviour and greater structural rearrangement over time. In contrast, the HSP90–MMs01053537 complex reached a relatively stable conformational regime much earlier in the simulation. After an initial rise to approximately 0.25–0.30 nm within the first few nanoseconds, the RMSD remained largely within a narrow window of about 0.26–0.35 nm for the remainder of the trajectory, with only minor fluctuations and a modest transient increase near the middle of the simulation. This more confined RMSD profile indicates that the MMs01053537-bound system underwent limited structural relaxation and maintained a comparatively consistent backbone arrangement under dynamic conditions.

The RMSF analysis further supported this trend, as shown in Figure 5b. In HSP90- geldanamycin complex, several residue segments exhibited higher local flexibility, with multiple peaks extending into the ∼0.10–0.19 nm range. These fluctuations were particularly evident in loop-rich regions and around the central portions of the protein, indicating greater residue-level mobility. On the other hand, the MMs01053537 complex showed a more restrained fluctuation pattern across most residues, with the majority of the backbone remaining below ∼0.05–0.07 nm and only a few localised regions showing modest increases. Importantly, the residue region around Arg173, which is relevant to the predicted interaction interface, appeared less mobile in the MMs01053537 complex than in the geldanamycin complex, supporting a more stable local environment in the hit-bound system.

The solvent accessible surface area (SASA) profiles showed that the HSP90–geldanamycin complex maintained a slightly lower overall solvent exposure, remaining around ∼119–123 nm^2^ with minor time-dependent variation. The HSP90–MMs01053537 complex, in contrast, remained at a somewhat higher SASA level, approximately ∼125–129 nm^2^, but still exhibited a stable trajectory with limited temporal drift. This suggests that the two ligands may induce slightly different surface exposure patterns during binding, while neither system showed evidence of major solvent-driven destabilization (Figure 5c).

The radius of gyration (Rg) analysis indicated that the HSP90–MMs01053537 complex was more compact than the geldanamycin-bound system. The geldanamycin complex showed a broader Rg profile, declining from approximately 1.85 nm–∼1.75 nm early in the simulation and then fluctuating between ∼1.72 and 1.78 nm for much of the trajectory. By contrast, the MMs01053537 complex remained more tightly distributed, with Rg values centered around ∼1.62–1.66 nm and only minor fluctuation throughout the simulation. This narrower and lower Rg distribution indicates a more compact and conformationally restrained protein–ligand assembly in the presence of MMs01053537 as shown in Figure 5d.

**FIGURE 6:**
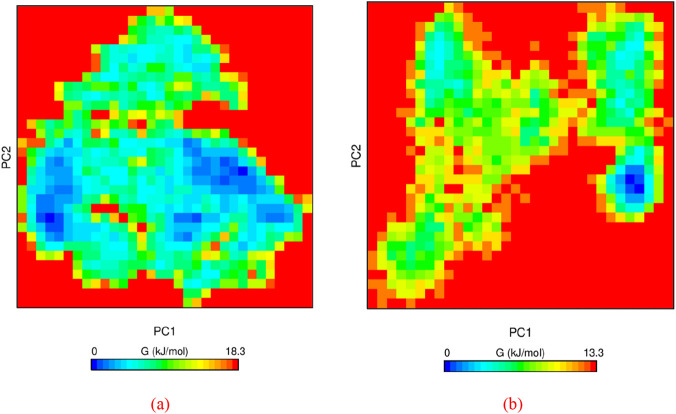
2D Gibbs free energy landscape of **(a)** HSP90–Geldanamycin (reference) complex **(b)** HSP90–MMs01053537 (hit) complex.

The Gibbs free-energy landscape (FEL) analysis as shown in Figure 6, revealed that the HSP90-reference complex sampled multiple low-energy conformational substates distributed across the PC1–PC2 conformational space. Several interconnected low-energy basins were observed rather than a single sharply confined minimum, indicating that the system underwent moderate conformational transitions during the simulation trajectory. The dominant conformational regions remained clustered within a relatively continuous energy distribution, suggesting that the complex maintained structural persistence while retaining conformational flexibility. Substantially, the HSP90–hit complex revealed the presence of multiple energetically favourable conformational basins distributed across the PC1–PC2 conformational space. The trajectory predominantly occupied a limited number of low-energy substates, indicating that the complex sampled several metastable conformations during the simulation period rather than undergoing unrestricted conformational drift. The presence of a distinct dominant low-energy basin together with a few additional local minima suggested that the hit-bound complex retained conformational adaptability while remaining within a relatively restricted energetic landscape.

### 3.7 Ensemble docking and binding free assessment from MD trajectories

The docking score of the HSP90–reference complex demonstrated stable interaction behaviour throughout the simulation trajectory, which ranged from −76.23 to −77.44 kcal/mol, yielding a mean docking score of −77.08 ± 0.43 kcal/mol whereas HSP90–hit complex remained consistently favourable across all trajectory-derived conformations, ranging from −85.34 to −85.93 kcal/mol, with a mean score of −85.65 ± 0.25 kcal/mol as shown in Table 4. Similarly, the binding free-energy estimates exhibited minimal variation, ranging from −7.01 to −7.16 kcal/mol, with an average ΔG value of −7.11 ± 0.05 kcal/mol for the HSP90-hit complex, whereas binding free-energy estimates ranged from −5.83 to −5.25 kcal/mol, with an average ΔG value of −5.58 ± 0.25 kcal/mol (Table 5).

**TABLE 4:** Docking scores of the reference molecule (Geldanamycin) and the hit molecule (MMs01053537) with HSP90 at different simulation time frames using Molsoft

S. No	Simulation time (ns)	References molecule*	Hit molecule^#^
1	0	−77.24	−85.92
2	20	−77.10	−85.34
3	40	−77.23	−85.42
4	60	−77.17	−85.56
5	80	−77.44	−85.77
6	100	−77.32	−85.93

* Margin of error = −–77.08 ± 0.40 kcal/mol.

^#^ Margin of error = −85.65 ± 0.23 kcal/mol.

**TABLE 5:** Binding free energy of the reference molecule (Geldanamycin) and the hit molecule (MMs01053537) with HSP90 at different simulation time frames using PRODIGY.

S. No	Simulation time (ns)	References molecule*	Hit molecule^#^
1	0	−5.79	−7.11
2	20	−5.56	−7.16
3	40	−5.31	−7.01
4	60	−5.25	−7.15
5	80	−5.78	−7.14
6	100	−5.83	−7.13

* Margin of error = −5.65 ± 0.24 kcal/mol.

^#^ Margin of error = −7.11 ± 0.05 kcal/mol.

## 4 Discussion

The present study identified novel peptidomimetics that disrupt the HSP90–HOP interface using a structure-guided computational pipeline. Earlier investigations primarily focused on identifying ligands capable of binding the HOP tetratricopeptide repeat (TPR) domains or the C-terminal MEEVD motif of HSP90 to prevent complex formation. Although the canonical HSP90–HOP interaction involves TPR2A docking to the C-terminal MEEVD motif, multiple studies have shown HOP can additionally engage N-terminal regions and thereby inhibit N-terminal dimerisation (Richter et al., 2003; Onuoha et al., 2008). In particular, hydrogen-exchange mass spectrometry and crosslinking analyses indicated that HOP stabilizes HSP90 in an open conformation, with its inhibitory effect involving not only the canonical C-terminal MEEVD interaction but also additional contacts that influence N-terminal dimerisation (Lee et al., 2012). Recent studies have also indicated that HOP binding extends beyond a simple anchor-contact model and may involve additional contacts and broader allosteric regulation of the HSP90 conformational cycle (Prodromou and Bjorklund, 2022; Bhattacharya and Picard, 2021). Therefore, the present study adopts a structure-guided computational design strategy in which interface-derived peptidomimetic candidates generated based on residues identified from the predicted HSP90–HOP docking interface. The integration of protein–protein docking, peptide modelling, multi-parameter screening integrated with machine learning, and molecular dynamics simulation enabled systematic prioritisation of candidates with favourable stability and interaction profiles.

The HSP90–HOP interaction represents a challenging yet pharmacologically relevant PPI interface for inhibitor discovery. Since PPIs generally involve broad and dynamic interaction surfaces, accurate identification of druggable subpockets is essential before docking and virtual screening studies. In the present study, Schrödinger SiteMap was employed to identify and characterize potential binding pockets within the HSP90 interface region involved in HOP recognition. SiteMap has been reported to effectively identify druggable regions within protein–protein interfaces (Lin et al., 2026). Importantly, the obtained DScore for the identified pocket indicated moderate-to-good druggability, which is particularly meaningful for PPI targets because such interfaces generally possess lower druggability scores than classical enzyme active sites. Subsequent protein–protein docking was carried out using PyDock, which generated multiple binding poses, which were further examined through interface residue analysis using PDBSum Generate. Notably, the top-ranked docking pose corresponded closely with the highest-scoring SiteMap pocket, indicating strong agreement between binding-site prediction and docking-based interface recognition as shown in Figure 2b. The consistency observed between SiteMap predictions, PyDock scoring, and interaction analysis strengthens the reliability of the proposed HSP90–HOP binding mode and supports the validity of the downstream computational investigations performed in this study.

**FIGURE 7:**
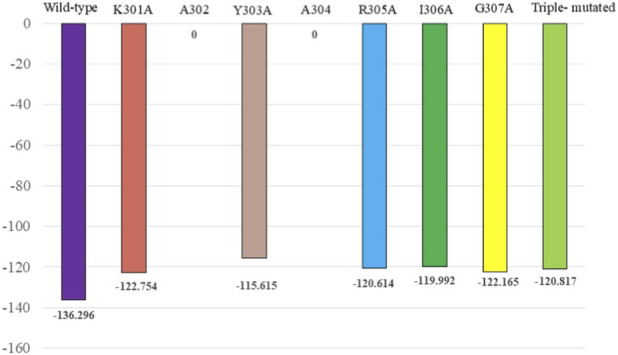
Graphical comparison of dock scores (kcal/mol) between wild–type and mutated peptides evaluated using the pyDockWEB server.

Interface residues from the complex were used to initiate peptide design, and the resulting sequence was iteratively optimised through molecular docking to improve binding affinity, ultimately yielding the candidate peptide p-KAYARIG. Although Ala304, Arg305 and Asp334 represent key interacting residues, peptide-based inhibitor design requires a continuous stretch of residues to preserve local secondary structure and enable realistic backbone conformations. Therefore, a contiguous peptide segment encompassing residues Lys301- Gly307 of HOP was selected as the template for peptide engineering. Furthermore, selecting a continuous segment allows preservation of the spatial orientation of the hotspot residues and facilitates subsequent structural modelling and docking analyses (Agoni et al., 2025). This truncation strategy was intentionally adopted to reduce unnecessary conformational flexibility and improve structural tractability during peptide docking and simulation, consistent with established approaches in protein–peptide interaction modelling and minimal motif-based inhibitor design (Ciemny et al., 2018). While additional interface contacts were observed elsewhere on the HOP surface, these interactions appeared less consistent across docking poses and were likely transient. In contrast, the selected region displayed persistent interface contacts in the top-ranked docked complexes, supporting its suitability as a template for peptidomimetic design. Our analysis corroborates these findings by identifying auxiliary N-terminal contacts in the modelled complex. Complementary site-directed computational mutagenesis identified Tyr303, Arg305, and Ile306 within the peptide as key determinants of interaction, as shown in Figure 7. These residues were used to retrieve a focused set of 200 peptidomimetic compounds for further evaluation. However, these residues should be regarded as computationally predicted contributors to binding rather than experimentally validated interaction determinants, and their functional roles require confirmation through further biochemical and structural investigations. Traditional machine learning algorithms continue to provide reliable and interpretable predictive performance in virtual screening, particularly when supported by high-quality curated datasets and informative molecular descriptors (Kumar and Acharya, 2022). The observed predictive performance by our in-house ML model is consistent with previous computational drug discovery studies demonstrating that conventional machine learning approaches remain effective for virtual screening applications (Srinivas et al., 2018). Furthermore, external prediction on structurally distinct peptidomimetic compounds demonstrated that the developed model retained satisfactory generalizability beyond the training chemical space, supporting its applicability for prospective compound prioritisation.

**FIGURE 8:**
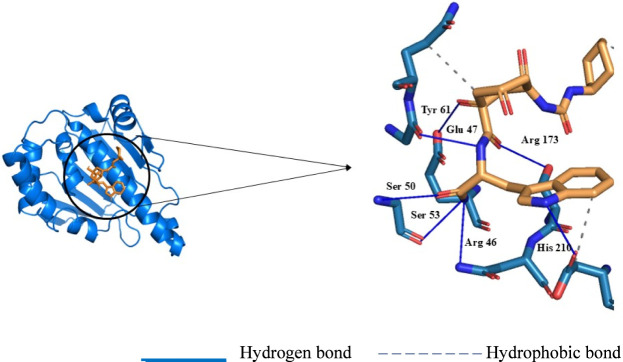
Docked conformation of HSP90 (blue) in conjunction with the lead peptidomimetic molecule (orange) shown in stick representation.

Notably, MMs01053537 exhibited a stable interaction with the residue Arg173, a residue predicted as indispensable for the direct molecular interaction between HSP90 and HOP (Figure 8). The engagement of this residue provides critical mechanistic support for the molecule’s potential to disrupt the HSP90–HOP interface, a target of interest in proteotoxic modulation. The predicted docking scores and scoring metrics further support its potential as a hit molecule. The hit molecule demonstrated a docking score of −85.92 kcal/mol, suggesting favourable binding characteristics within the docking analysis. Additionally, the compound also exhibited the most favourable binding free energy more than the reference compounds (ΔG = −7.11 kcal/mol) among all shortlisted candidates as predicted by the PRODIGY server. The compound exhibited stronger non-electrostatic stabilisation and a much denser electrostatic interface, supporting the likelihood of a more stable interaction. Recent advances in machine-learning–based scoring have markedly improved virtual screening reliability. Lower scores in KDEEP, SF-CNN, and X-Score for ligand compounds also support higher affinity for the receptor (Murali and Karuppasamy, 2022). The complementary scoring functions also produced consistent results, with the hit compound showing a KDEEP score of −13.8295 kcal/mol, an SF-CNN score of 9.3973 kcal/mol, and an X-score of −7.05 kcal/mol. These scoring functions were originally trained predominantly on protein–small molecule complexes. However, peptidomimetic compounds often share structural and physicochemical characteristics with small-molecule ligands, allowing these models to provide useful estimates of relative binding propensity (Lenci and Trabocchi, 2020).

The ADMET properties of the shortlisted peptidomimetic candidates were evaluated as part of the early-stage computational screening pipeline to support compound prioritization. These predictions were interpreted as preliminary *in silico* estimates of pharmacokinetic and toxicity-related properties, including oral bioavailability, gastrointestinal absorption, and potential hepatotoxicity, rather than definitive indicators of *in vivo* behaviour. In particular, predicted non-DILI status should not be considered evidence of toxicological safety, but rather as a comparative indication of lower predicted hepatotoxic risk within the constraints of the computational model. While many modern peptidomimetics are structurally optimized to improve pharmacokinetic properties and are often designed to comply with or closely approximate Lipinski’s rule of five, such compliance should be interpreted as a preliminary indicator of physicochemical suitability rather than definitive evidence of drug-likeness. Accordingly, the ADMET analysis performed in this study was used as an early-stage computational filtering strategy to support candidate prioritization, whereas experimental pharmacokinetic, permeability, and toxicological studies remain necessary for further validation. In our study, the hit compound was filtered and refined to meet Ro5 criteria, ensuring favourable ADME properties. Given its continued relevance, Ro5 served as a key early-stage filter to ensure drug-likeness in our rationally designed peptidomimetic (Lau and Dunn, 2018). A recent study by Ardestani et al. (2022) demonstrated that 1,2,4-triazole rings, a crucial component of our lead compound, can effectively disrupt HSP90 function, thereby validating the appropriateness of our scaffold selection. Similarly, Chandrasekaran et al., 2025 reported that the fused triazole systems, such as triazolo-pyridines and triazolo-pyrimidines, adopt a planar conformation that allows them to fit precisely within the nucleotide-binding pocket of HSP90. Several derivatives have also exhibited sub-micromolar IC_50_ values in ATPase assays and have induced client protein degradation in cancer cells, underscoring the potential of these scaffolds to modulate HSP90 function and downstream oncogenic processes. Furthermore, indole derivatives present in our hit compound are widely regarded as privileged scaffolds in the design of HSP90 inhibitors (Abdullah and Omran, 2024). These structural features not only corroborated the binding potential of our hit molecule but also suggest dual-targeting capabilities that may enable disruption of the HSP90–HOP interaction in addition to ATP-competitive inhibition. Collectively, these studies substantiate the structural and functional basis of our peptidomimetic molecule and validate its potential to inhibit HSP90, potentially through dual mechanisms involving ATP competition and disruption of the HSP90–HOP protein-protein interaction.

The PASS server has demonstrated reliable predictive performance across diverse compound classes, providing Pa/Pi probability scores that effectively flag likely bioactivities and toxicities for early-stage hit identification. Compounds were considered more likely to exhibit the predicted biological activity when Pa exceeded Pi, with higher Pa values indicating greater predicted probability of activity and lower Pi values indicating reduced predicted probability of inactivity. The hit molecule was predicted to exhibit potential antagonistic activity against HSP27, which plays a crucial role in conferring resistance to chemotherapy and radiation by blocking apoptotic pathways. Recent studies have highlighted the therapeutic promise of targeting HSP27. For instance, the suppression of HSP27 in glioblastoma cells restored their sensitivity to temozolomide and induced apoptotic cell death (Lampros et al., 2022). HOP regulates the conformational cycle and restrains the progression towards the ATP-dependent client-maturation state by engaging both of its N-terminal and C-terminal. This regulatory role is tightly connected to the behaviour of small heat-shock proteins such as HSP27, whose expression, oligomeric state, and chaperone function are governed by HSP90-dependent client signalling pathways and stress-response networks. Consequently, perturbation of the HSP90–HOP interaction may indirectly influence downstream chaperone signalling pathways. The PASS-derived HSP27-related activity is presented solely as a preliminary computational bioactivity prediction generated by the PASS algorithm and should not be interpreted as validated evidence of direct HSP27 targeting or mechanistic involvement. However, this prediction does not provide structural or mechanistic evidence for direct inhibition of HSP27 by the identified compound, and therefore the association should be interpreted cautiously pending further experimental validation (Wang et al., 2014). Further dedicated experimental and structural studies would be required to evaluate any potential interaction of the compound with HSP27-associated pathways, representing an interesting avenue for future work to further substantiate these findings (Zhao et al., 2024).

The comparative MD analysis provided useful insight into the relative stability of the HSP90–MMs01053537 and HSP90–geldanamycin complexes. RMSD analysis demonstrated that the geldanamycin-bound complex underwent comparatively larger structural deviations throughout the simulation, including multiple transient conformational excursions and late-stage increases in backbone displacement. Such behaviour suggested continued structural rearrangement and comparatively higher conformational flexibility within the reference system. The MMs01053537-bound complex underwent limited conformational relaxation and then remained within a stable fluctuation window, compared to the geldanamycin-bound complex. In the context of MD simulations, such behavior is often interpreted as greater conformational restraint and structural persistence for the former complex. This observation was reinforced by the lower Rg values observed for HSP90–MMs01053537, indicating a more compact architecture and reduced tendency toward structural expansion during the simulation (Paranthaman et al., 2025).

The residue-level RMSF profile further suggested that MMs01053537 conferred a more restrained dynamic environment across much of the HSP90 backbone. Lower RMSF values generally reflect reduced local flexibility and can indicate a more stable protein–ligand arrangement, particularly when observed consistently across the majority of residues. By contrast, the geldanamycin complex showed more pronounced fluctuations in several regions, implying greater local mobility. Importantly, these fluctuations did not indicate instability or dissociation, but they do suggest that the MMs01053537 complex may better preserve a stable interaction environment under dynamic conditions (Chahir et al., 2026). This further confirmed that MMs01053537 imposed a global rigidification of HSP90, with particular emphasis on key loop and predicted interface regions (Chen et al., 2018). The slight increased SASA for the hit indicated that MMs01053537 stabilised an open but rigidified conformation. Such conformational plasticity may be advantageous for allosteric regulation, as reported for other HSP90 inhibitors that lock the N-terminal domain in distinct states conducive to client release (Ghosh et al., 2025). In this case, the modestly higher SASA of the MMs01053537 complex may reflect a different binding pose or interface arrangement rather than loss of stability, especially since the same complex simultaneously showed lower RMSD, lower Rg, and reduced RMSF. Thus, the SASA profile should be considered alongside the other structural descriptors rather than in isolation.

The Rg analysis additionally indicated that the MMs01053537-bound system maintained a more compact structural organization throughout the simulation period. The geldanamycin complex exhibited broader Rg fluctuations and substantial early-stage compaction, consistent with greater conformational adaptation and global structural rearrangement during equilibration. In contrast, the comparatively narrow Rg distribution observed for the HSP90–MMs01053537 complex suggested maintenance of a compact and conformationally stable ensemble. Since Rg reflects the overall spatial distribution of atomic mass around the molecular center of mass, reduced fluctuation is often associated with greater structural compactness and conformational persistence in protein–ligand systems (Paranthaman et al., 2025).

Free-energy landscape analysis provides insight into the conformational behavior and metastable states sampled by biomolecular systems during molecular dynamics simulations. The HSP90-reference profile demonstrated a broader and more continuous distribution of low-energy conformations extending across multiple regions of the conformational landscape. The existence of several interconnected metastable basins suggests that the system underwent greater conformational exploration and sampled a wider range of structural substates during the simulation (Murali and Karuppasamy, 2023). The HSP90–hit complex behavior is generally consistent with a dynamically stable protein–ligand complex that retains conformational flexibility without exhibiting major structural destabilization. The presence of multiple metastable minima suggested that the complex underwent moderate conformational adaptation during the simulation, which is expected for flexible protein systems such as HSP90. Importantly, the absence of extensive high-energy dispersion or highly fragmented conformational sampling indicated that the bound complex did not experience substantial conformational instability during the simulation period. The observed conformational heterogeneity may also reflect adaptive stabilisation of the ligand-bound interface under dynamic conditions rather than a single rigid binding mode. Similar metastable free-energy basins have frequently been associated with structurally persistent yet flexible biomolecular interaction systems in molecular dynamics studies (Padhi et al., 2022). Therefore, the FEL profile obtained in the present study further supported the dynamic persistence of the predicted HSP90–hit complex and complemented the other trajectory analyses. Overall, the stability analysis conducted through MD simulation supports our claim that MMs01053537 is a putative disruptor of the HSP90–HOP interaction.

Traditional docking approaches may not adequately account for the flexibility and conformational adaptation of the receptor occurring during molecular dynamics simulations. Therefore, ensemble docking and MD-enhanced binding free energy analyses were incorporated into the present study to evaluate whether the predicted HSP90 interaction profiles remained stable across multiple trajectory-derived conformations rather than depending on a single static docking pose. The comparatively stable docking and binding free-energy values observed throughout the MD trajectory suggested that both the peptide and hit compound retained favourable interaction profiles under dynamic simulation conditions. In particular, the low variability observed in the ensemble-derived docking scores and binding free energy analysis indicated that the predicted binding modes were not substantially disrupted by conformational fluctuations occurring during the simulation period (Amaro et al., 2018). This consistency supported the structural persistence of the proposed interaction environment and suggested that the identified complexes maintained stable interface complementarity throughout the trajectory.

Importantly, the MD-enhanced binding free-energy analysis was interpreted as a comparative contact-based affinity assessment rather than as an absolute thermodynamic free-energy calculation, since the PRODIGY framework employed regression-based interface descriptors derived from intermolecular contact composition and non-interacting surface properties. Furthermore, the incorporation of statistical analyses, which included standard deviation, standard error, and confidence interval estimation, improved the interpretability and robustness of the ensemble-derived computational data. The low statistical variability observed across the extracted MD conformations supports the reproducibility of the computational workflow and indicates that the interaction profiles were not strongly dependent on isolated trajectory snapshots. Collectively, these findings support the dynamic stability of the proposed complexes (Ziemann et al., 2023).

Recent advances in computational drug discovery have increasingly incorporated heterogeneous biological network integration, geometric deep learning, motif-aware attention mechanisms, and three-dimensional molecular representation learning to improve biomolecular interaction prediction and compound prioritisation (Zhao et al., 2024; Zhang et al., 2026). Contemporary frameworks combining graph-based learning, multi-source biological interaction modelling, and structure-aware neural architectures have demonstrated improved predictive robustness and interpretability across diverse drug discovery applications (Zhao et al., 2022). Similarly, the integration of three-dimensional molecular spatial representations with multi-perspective learning strategies has further highlighted the growing importance of data-driven and structurally informed computational methodologies in modern therapeutic discovery pipelines. In this context, the present computational framework integrates structure-based docking, ML-assisted rescoring, ensemble docking, MD-enhanced binding free energy estimation, and statistical evaluation to support the identification and prioritisation of potential HSP90–HOP interaction modulators. While the present findings remain computational and require experimental validation, the proposed workflow provides a rational framework for the identification and prioritisation of potential peptidomimetic modulators targeting the HSP90–HOP interaction, thereby contributing incremental insights into strategies aimed at disrupting this chaperone complex. Furthermore, the future integration of advanced representation-learning and network-based computational methodologies may further enhance the scalability, predictive robustness, and translational applicability of subsequent iterations of the workflow.

## 5 Conclusion

The absence of potent peptidomimetic inhibitors targeting the HSP90–HOP interface underscored a significant gap in modulating proteostasis during tumour invasion. In this study, MMs01053537 was identified as a promising peptidomimetic candidate and was hypothesised to disrupt the HSP90–HOP interaction. The molecule exhibited favourable interactions with HSP90, along with robust stability profiles during MD simulations. The presence of key structural moieties, such as triazole and indole rings, significantly enhanced the anti-proliferative activity reported in the literature, highlighting their potential pharmacological relevance. These results collectively identified MMs01053537 as a prospective candidate for disrupting the HSP90–HOP interaction. Experimental validation represents an important future direction to confirm the biological relevance of the predicted interactions and therapeutic utility of the hit compound.

## Data Availability

The code corresponding to classification model is publicly available in GitHub repository: https://github.com/vitmilab/HSP90_Activity_prediction.git.
